# Microstructure, Mechanical Properties, and Corrosion Resistance of Ag–Cu Alloys with La_2_O_3_ Fabricated by Selective Laser Melting

**DOI:** 10.3390/ma16247670

**Published:** 2023-12-15

**Authors:** Xueyang Zhao, Haiyan Zheng, Xin Ma, Yinying Sheng, Dahai Zeng, Junping Yuan

**Affiliations:** 1 Jewelry Institute, Guangzhou Panyu Polytechnic, No. 1342 Shiliang Road, Guangzhou 511483, China; zhaoxy@gzpyp.edu.cn (X.Z.); 13067263229@163.com (H.Z.); 13138598763@163.com (X.M.); yuanjp@gzpyp.edu.cn (J.Y.); 2 Institute of Corrosion Science and Technology, No. 136 Kaiyuan Avenue, Guangzhou 510530, China; 3 Institute of Advanced Wear & Corrosion Resistant and Functional Materials, Jinan University, No. 601 Huangpu Avenue West, Guangzhou 510632, China

**Keywords:** Ag alloy, La_2_O_3_, selective laser melting, electron back scatter diffraction

## Abstract

Ag and its alloys, when prepared by a selective laser melting (SLM) process, have a low density and poor overall performance due to their high reflectivity when the most commonly used laser (*λ* = 1060 nm) is used, and they have exorbitant thermal conductivity. These characteristics lead to the insufficient melting of the powders and severely limit the applications of additive manufactured silver alloys. To improve the absorption of the laser, as well as for better mechanical properties and higher resistance to sulfidation, Ag–Cu alloys with different La_2_O_3_ contents were prepared in this work using the SLM process, via the mechanical mixing of La_2_O_3_ nanoparticles with Ag–Cu alloy powders. A series of analyses and tests were conducted to study the effects of La_2_O_3_ in Ag–Cu alloys on their density, microstructure, mechanical properties, and corrosion resistance. The results revealed that the addition of La_2_O_3_ particles to Ag–Cu alloy powders improved the laser absorptivity and reduced defects during the SLM process, leading to a significant rise from 7.76 g/cm^3^ to 9.16 g/cm^3^ in the density of the Ag–Cu alloys. The phase composition of the Ag–Cu alloys prepared by SLM was Silver-3C. La_2_O_3_ addition had no influence on the phase composition, but refined the grains of the Ag–Cu alloys by inhibiting the growth of columnar grains during the SLM process. No remarkable preferred orientation existed in all the samples prepared with or without La_2_O_3_. An upwards trend was achieved in the hardness of the Ag–Cu alloy by increasing the contents of La_2_O_3_ from 0 to 1.2%, and the average hardness was enhanced significantly, from 0.97 GPa to 2.88 GPa when the alloy contained 1.2% La_2_O_3_ due to the reduced pore defects and the refined grains resulting from the effects of the La_2_O_3_. EIS and PD tests of the samples in 1% Na_2_S solution proved that La_2_O_3_ addition improved the corrosion resistance of the Ag–Cu alloys practically and efficaciously. The samples containing La_2_O_3_ exhibited higher impedance values and lower corrosion current densities.

## 1. Introduction

As a type of common noble metal, silver has been used in ornaments and coins for hundreds of years, and is still popular in jewelry and electrical contact materials [1,2]. Pure silver exhibits low strength and hardness [3], making it prone to scratches and wear scars on its surface. The inferior mechanical properties of silver can downgrade its luster and decorative function in jewelry, and also limits its application in electrical contacts [4,5]. Alloying is a simple and effective method for improving the performance of pure metals [6,7]. Among the numerous available alloys [8,9,10], Ag–Cu alloys [11,12] are a commonly used silver alloy for electrical contacts and decoration purposes, due to its improved mechanical performance, good electrical and thermal conductivity, and moderate cost. However, the introduction of the Cu element reduces the Ag’s chemical stability, such that the Ag–Cu alloy is more likely to react with the element sulfur to generate black sulfides, in comparison to pure silver [13]. The existence of sulfides can also negatively affect the functionality of silver jewelry and the conductivity of electrical components [14]. Additionally, due to the alloy’s two-phase structure at room temperature, micro-batteries generated at the phase interface in the corrosion medium would accelerate the corrosion behavior of the Ag–Cu alloy [15]. Thus, it is necessary to further optimize the composition of the Ag–Cu alloy by combining the two components’ excellent mechanical properties and corrosion resistance, including sulfidation resistance [16,17].

Development trends in the customization of jewelry, as well as in the miniaturization and complexity of electrical components, have attracted increasing attention from researchers [18,19]. While many metallic products produced by additive manufacturing have been extensively applied in many fields [20,21,22], there are only a few research studies on the preparation of silver or its alloys by laser powder bed fusion technology. Most reports have remained stuck on the preliminary exploration of process parameters [4,18]. Besides the high cost of silver powder, the more important reason for this is that the high reflectivity to the lasers used (*λ* = 1060 nm) and the exorbitant thermal conductivity [23,24] of silver leads to insufficient melting of the powders during laser scanning. As such, the silver products produced by additive manufacturing usually have a low density and poor overall performance. To reduce defects and improve the density of the finished product, two feasible solutions have been proposed by researchers. The first one focuses on the laser equipment and process parameters; a shorter-wavelength laser, higher laser power, and smaller laser-focused spots can be used to increase the absorption and energy density of the laser, which make the powder melt better [25,26,27]. The other method concerns the powders; it has been proven by some studies that adding alloying elements or mixing particles or fibers with other powders could reduce the reflectivity of silver effectively [26,28,29].

Inspired by previous works, La_2_O_3_ nanoparticles were mixed in Ag–Cu alloy powders in this work. La_2_O_3_ is a kind of rare earth oxide (REO) often-used in the preparation of steel [30], glass [31], and ceramics [32]. REO was used in this work mainly based on the following three considerations: Firstly, rare earth ions are able to absorb laser rays over a wide wavelength range because of their special 4 f electronic configuration [33,34], which is able to increase the laser absorptivity when added into Ag–Cu alloy powders. Secondly, rare earth elements have often been applied in metallurgical fields because of their significant effects on refining the grain, purifying the grain boundary, and microalloying [35,36,37], etc. This is a feasible method for improving the mechanical properties of Ag–Cu alloys. Finally, rare earth elements are helpful for decreasing the generation of sulfide in silver alloys according to some research studies [14,38]. For the above reasons, Ag–Cu alloys with different contents of La_2_O_3_ were prepared by a selective laser melting (SLM) process in this work. Then, to verify the effects of the content of La_2_O_3_ in selective-laser-melted silver alloys on their phase compositions, microstructures, mechanical properties, and corrosion resistance in Na_2_S solution, a series of studies were conducted in detail.

## 2. Materials and Methods

### 2.1. Preparation of Ag–Cu Alloys with Different La_2_O_3_ Contents by SLM

**Figure 1:**
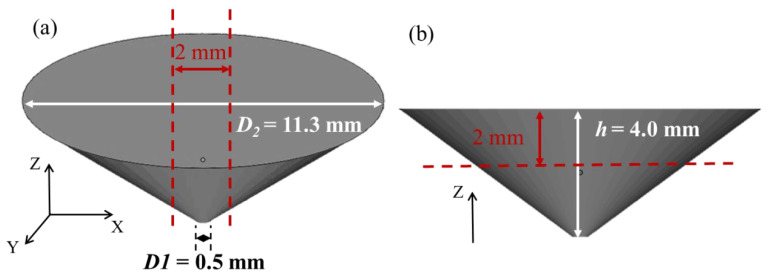
The specific sizes of the Ag–Cu alloy sample model: (**a**) isometric view and (**b**) front view.

**Figure 2:**
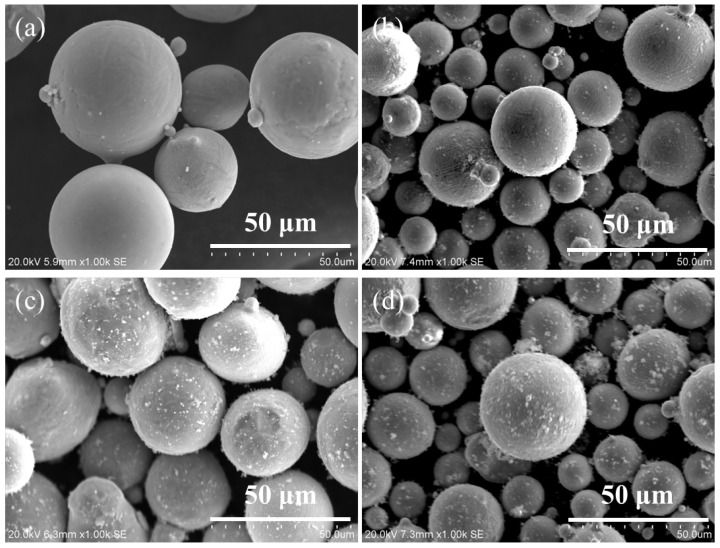
The SEM images of the Ag–Cu alloy powders mixed with a content of (**a**) 0, (**b**) 0.4%, (**c**) 0.8%, and (**d**) 1.2% La_2_O_3_ particles.

The digital model of the Ag–Cu alloy was designed in a conical shape using Magics software (v20.03); the detailed dimensions of the sample are shown in Figure 1. The Ag–Cu alloy powders used in this work were supplied by Legor Group, and the chemical compositions of the powders analyzed by an X-ray fluorescence spectrometer are listed in Table 1. The different contents (mass fraction of 0, 0.4%, 0.8% and 1.2%) of La_2_O_3_ particles, with a purity of more than 99% and a diameter of less than 1 μm, were uniformly mechanically admixed in Ag–Cu alloy powders by a drum mixer (shown in Figure 2). A Sisma Mysint 100 SLM system with a Nd: YAG fiber laser (that the wavelength was 1060 nm) was employed for the preparation of the designed samples. The parameters applied in the present work were dependent on the author’s previous work, as shown in Table 2.

### 2.2. Characterizations

After the samples were prepared by the SLM process, the samples were cut into flat pieces with the thickness of 2 mm along the dashed line, as shown in Figure 1, for further investigation into the differences between the XY and Z planes, separately. An X-ray diffraction (XRD, Bruker D8 Advance, Bruker Corporation, Billerica, MA, USA) system was employed to identify the phase compositions of the Ag–Cu alloy samples prepared by SLM using a Cu Kα radiation source in θ/2θ scanning mode; scanning ranged from 20° to 90° at a rate of 5°/min. The densities of the samples with different La_2_O_3_ contents were measured by a DX-300 density tester, and the average density of three samples of the same content was calculated to ensure accuracy. The mixed powders and defects after the preparation of the samples with different La_2_O_3_ contents were observed by a Hitachi S3400 N scanning electron microscope (SEM). The distribution mapping of Ag and La in the Ag–Cu alloy prepared by SLM was corroborated with an electron probe micro analyzer (EPMA, JXA-iHP200F). Moreover, the details of the samples concerning their microstructure and preferred orientation were analyzed by a Thermo Scientific Apreo 2 SEM equipped with electron back scatter diffraction (EBSD). Before being analyzed by EBSD, the samples were ion beam-polished to meet the requirements.

### 2.3. Mechanical Properties

An Agilent Nano Indenter with a diamond Berkovich tip was used to measure the hardness of the samples with varying La_2_O_3_ contents. The tests were carried out under the conditions of a maximum load of 30 g, a loading/unloading rate of 30 mN/s, a peak holding time of 10 s, and a Poisson’s ratio of 0.36, respectively. To ensure the accuracy of the resultant data, at least 5 individual indentation tests were conducted for every sample.

### 2.4. Corrosion Behavior in Na_2_S Solution

The existence of sulfides in silver and its alloys negatively affects its conductivity and decorative performance. The corrosion resistance performance of selective laser-melted samples with different La_2_O_3_ contents in 1% (mass fraction) Na_2_S solution was evaluated using a PARSTAT 4000 electrochemical workstation with a standard three-electrode system. During the test, a platinum plate (the size of 10 × 10 × 0.2 mm) and samples with an exposure area of 1 cm^2^ served as the counter electrode and the working electrode, respectively. A saturated calomel electrode was chosen as the reference electrode. An electrochemical impedance spectroscopy (EIS) test and potentiodynamic (PD) polarization were conducted on the samples to assess the impact of adding La_2_O_3_ on the corrosion resistance. The measurements of EIS and PD for all the samples were conducted after achieving a stable open circuit potential (OCP). The EIS tests were carried out by applying an amplitude of 10 mV at the OCP and scanning frequencies from 100,000 to 0.01 Hz. The PD polarization analysis was executed from −0.3V to +0.3V vs. OCP using a scan rate of 1 mv/s.

## 3. Results and Discussion

### 3.1. The Effect of La_2_O_3_ Contents on the Densities and Phase Compositions of Ag–Cu Samples Prepared by SLM

**Figure 3:**
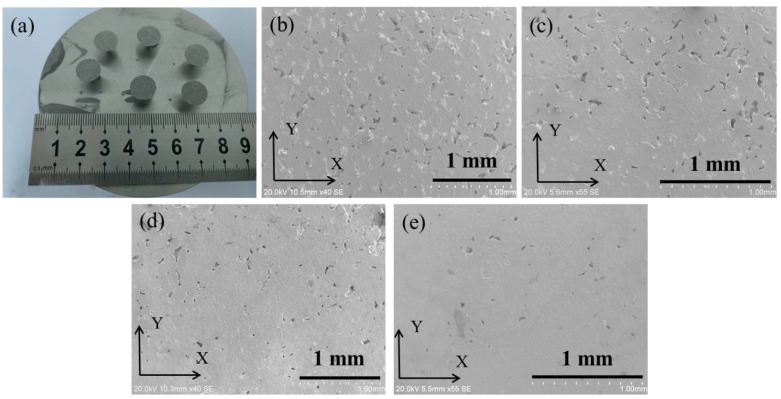
(**a**) Photos of the Ag–Cu alloys prepared by the SLM process, and the SEM images of the samples with La_2_O_3_ contents of (**b**) 0, (**c**) 0.4%, (**d**) 0.8%, and (**e**) 1.2%.

Conical Ag–Cu alloy samples with different La_2_O_3_ contents were successfully prepared using the SLM process. As shown in Figure 3a, the sizes of the prepared samples were consistent with the designed model. The samples presented a gray metallic luster and seemed to be relatively rough. From Figure 3b–e, various amounts of pore defects in the Ag–Cu alloy, caused by insufficient melting of the powders, could be seen in the XY plane. Furthermore, it is noteworthy that as the La_2_O_3_ content increased from 0 to 1.2%, there was a decline in the number of defects observed in the images. The results of the density measurements for the samples with different La_2_O_3_ contents, as listed in Table 3, also support this viewpoint; this indicates that the addition and mixture of La_2_O_3_ nano-particles to Ag–Cu alloy powders plays a positive role in improving laser absorption during the SLM process, leading to a significant increase in the density of the Ag–Cu alloys, rising from 7.76 g/cm^3^ to 9.16 g/cm^3^.

**Figure 4:**
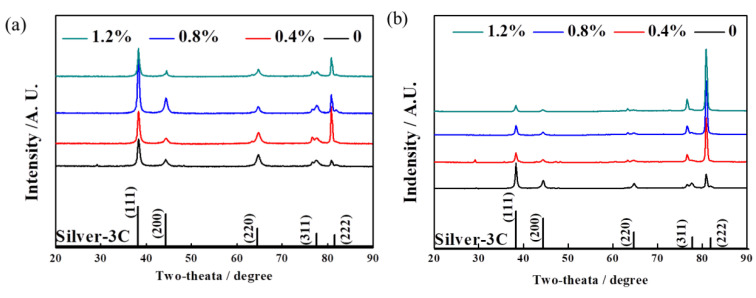
XRD patterns of SLMed Ag–Cu alloys with different La_2_O_3_ contents in the (**a**) XY and (**b**) Z plane.

To identify the phase compositions and to analyze the grain orientation, XRD tests were executed on both the XY and Z planes of the Ag–Cu alloys prepared using SLM with different La_2_O_3_ contents. The results are shown in Figure 4. Firstly, the phase composition of all the samples as-prepared was Silver-3C, and no diffraction peak of any other phase was observed. Secondly, the relative intensities of the diffraction peaks changed after the addition of La_2_O_3_. The intensities of the (222) crystal plane of the samples with La_2_O_3_ showed a significant increase in both the XY and Z planes, compared to the patterns of the samples without La_2_O_3_ addition and the data from the powder diffraction files (PDF) provided by the International Centre for Diffraction Data (ICDD), shown at the bottom in Figure 4. The (222) planes are parallel to the (111) planes, but the interplanar distance is different. The notable rise in the relative intensities of the (222) plane could be attributed to the distortion caused by La atoms diffused into the Ag lattices. Although the relative diffraction intensities of the (111) and (222) planes were higher than those of the PDF data, further research was necessary to confirm whether a preferred orientation existed in the selective laser-melted Ag–Cu alloys.

### 3.2. The Effect of La_2_O_3_ Content on the Microstructure of Ag–Cu Samples Prepared by SLM

**Figure 5:**
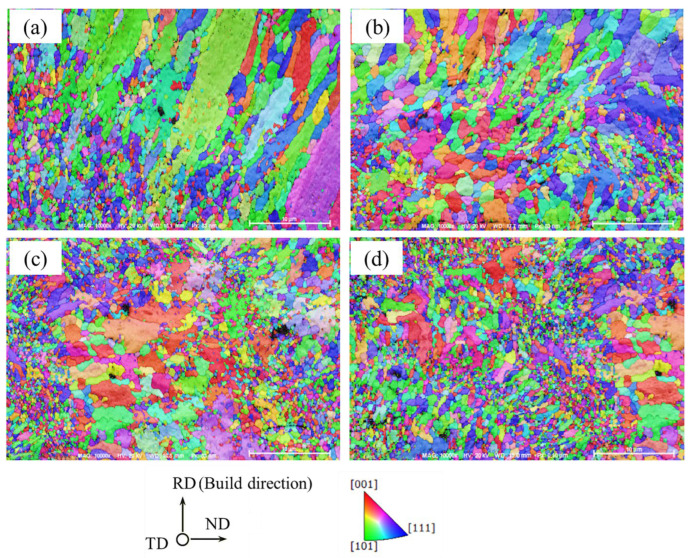
EBSD maps of SLMed Ag–Cu alloys with a La_2_O_3_ content of (**a**) 0, (**b**) 0.4%, (**c**) 0.8%, and (**d**) 1.2%, scale: 10 μm.

To investigate the microstructure characteristics of Ag–Cu alloys with varying La_2_O_3_ contents fabricated by SLM, EBSD technology was employed to analyze their grain size, morphologies, and orientation. As shown in Figure 5, the grain size and morphology were apparently influenced by the addition of La_2_O_3_. In the sample without La_2_O_3_, a number of large columnar grains were visible. The columnar grains were oriented parallel to the building direction, and there were also numerous fine equiaxed grains observed. These fine equiaxed grains were able to form at the border of the melting pool, where the cooling rate was higher than that in the middle area of the melting pool. With a slower cooling rate, the grains could grow larger in the direction of the temperature gradient. When 0.4% La_2_O_3_ was added, both the number and the length of the columnar grains decreased notably, while the ratio of equiaxed grains was enhanced. As the La_2_O_3_ content reached 0.8% and 1.2%, the microstructures were dominated by equiaxed grains. The transition of grain morphology indicated that the addition of La_2_O_3_ could inhibit the growth of columnar grains during the SLM process.

**Figure 6:**
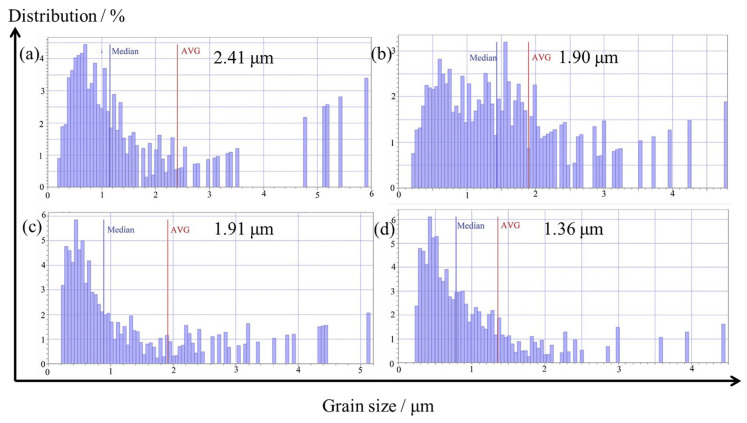
Average grains sizes of Ag–Cu alloys with different La_2_O_3_ contents of (**a**) 0, (**b**) 0.4%, (**c**) 0.8%, and (**d**) 1.2%.

The average grain sizes of the Ag–Cu alloys with different La_2_O_3_ contents, as calculated based on the observations in Figure 5, are presented in Figure 6. The average grains size of the Ag–Cu alloys without La_2_O_3_ was 2.41 μm, which was larger than that of the samples containing La_2_O_3_. As the contents of La_2_O_3_ increased from 0.4% to 0.8% and 1.2%, the corresponding grain size values decreased to 1.90 μm, 1.91 μm, and 1.36 μm, respectively. The average grain sizes of the sample with 0.4% and 0.8% La_2_O_3_ were quite close, but the sample with 0.8% La_2_O_3_ had a higher distribution of smaller grains compared to the sample with 0.4% La_2_O_3_. Based on the aforementioned results, the addition of La_2_O_3_ could refine the grains of Ag–Cu alloys prepared by SLM.

**Figure 7:**
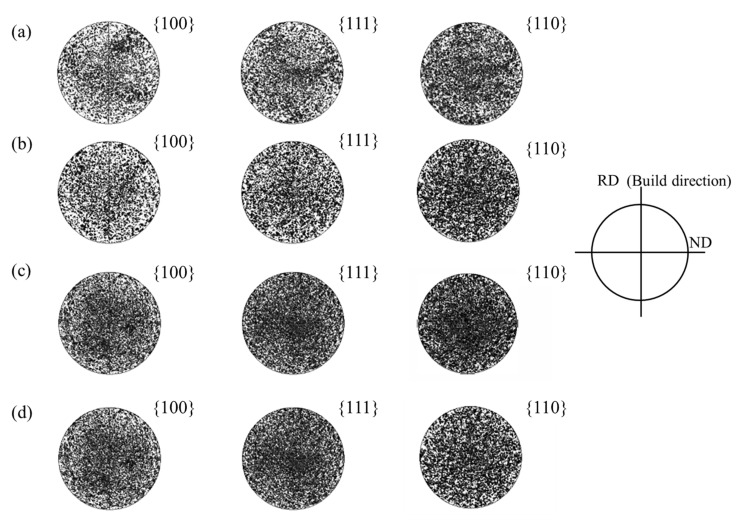
Pole figures of the Ag–Cu alloys prepared by SLM with a La_2_O_3_ content of (**a**) 0, (**b**) 0.4%, (**c**) 0.8%, and (**d**) 1.2%.

As mentioned previously, to confirm the presence of a preferred orientation in the selective laser melted Ag–Cu alloys and to investigate the effect of La_2_O_3_ on grain orientation, pole figures of Ag–Cu alloys with different La_2_O_3_ contents were obtained using EBSD technology. These pole figures are presented in Figure 7. The information presented in Figure 7 shows that no significant preferred orientation existed in either the samples prepared with or those without La_2_O_3_. This again shows that the notable rise in the relative intensities of the (222) plane in Figure 4 was caused by La atoms entering into the Ag lattices, rather than the preferred orientations being generated after La_2_O_3_ was added. These results might also be caused by the fact that the chosen area for magnification was too small to accurately represent the entire sample. However, due to the exceptionally small grain sizes of the sample caused by the high cooling rate of the process and the excellent thermal conductivity of Ag–Cu alloys, a higher magnification had to be chosen in order to reveal the details of their morphology.

**Figure 8:**
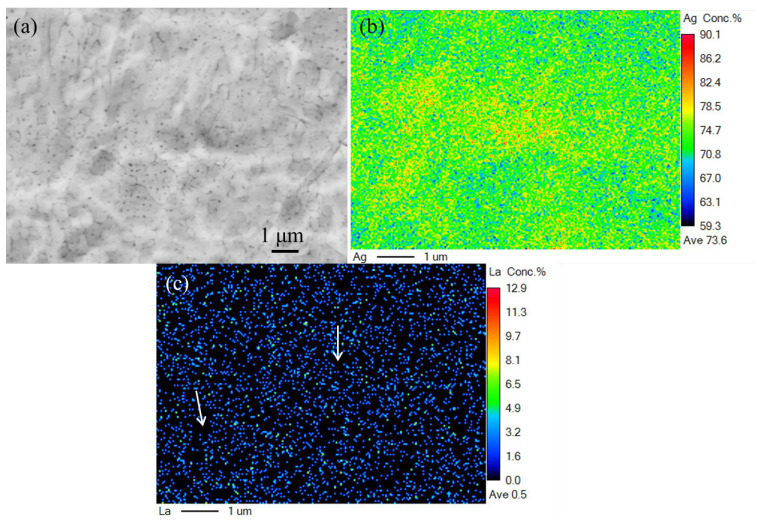
(**a**) The morphology, and elemental distribution of (**b**) Ag and (**c**) La in the sample with 1.2% La_2_O_3_.

The elemental mappings of Ag and La in the as-prepared Ag–Cu alloys were assayed using EPMA technology in this study. The sample containing 1.2% La_2_O_3_, which had the highest La_2_O_3_ content among all the samples, was chosen to determine whether the element of La concentrated at grain boundaries or within grains. As shown in Figure 8a, a number of fine and dense equiaxed grains and cellular grains were observed in the selected area, which was similar to the morphology shown in Figure 5d. The distribution of Ag was very uniform in Figure 8b, but La was barely present in some areas marked in Figure 8c. The specific areas appeared to be narrow sheets, resembling grain boundaries more. At this point, we have a comprehensive understanding of how the La_2_O_3_ content affects the microstructure of Ag–Cu samples produced through SLM.

### 3.3. The Effect of La_2_O_3_ Content on the Mechanical Properties of Ag–Cu Samples Prepared by SLM

**Figure 9:**
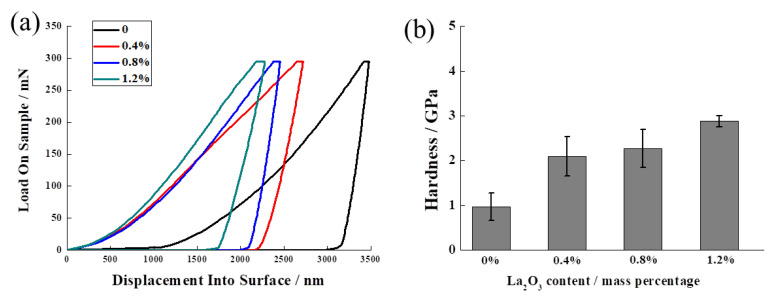
(**a**) The load and unload curves and (**b**) average hardness of Ag–Cu alloys with different La_2_O_3_ contents prepared by SLM.

To evaluate the mechanical properties of the Ag–Cu alloys with different La_2_O_3_ contents, the nano-indentation technique was used to measure the hardness of all the samples. The results are presented in Figure 9. As shown in Figure 9a, when a maximum load of 300 mN was applied on the samples using the tip, the indentation depths of the samples with La_2_O_3_ were found to be lower than that of the sample without La_2_O_3_. With an increase in La_2_O_3_ content from 0 to 1.2%, the indentation depths declined gradually at the max load. The residual indentation depth followed the same law during unloading, indicating that the deformation of the samples decreased as the La_2_O_3_ content decreased from 1.2% to 0.8%, 0.4%, and 0. The average hardness of the Ag–Cu alloys with different La_2_O_3_ contents, as depicted in Figure 9b, provided more direct results. The upward trend of the hardness was prominent with increasing La_2_O_3_ contents. The average hardness of Ag–Cu samples prepared by SLM without La_2_O_3_ was 0.97 GPa; it raised to 2.10 GPa, 2.27 GPa, and 2.88 GPa when 0.4%, 0.8%, and 1.2% La_2_O_3_ was mixed into the powders, respectively. The enhancement in the hardness of the Ag–Cu alloys was mostly due to reduced pore defects and the more refined grains created by the effects of La_2_O_3_.

### 3.4. The Effect of La_2_O_3_ Content on the Corrosion Resistance of Ag–Cu Samples Prepared by SLM in 1% Na_2_S Solution

**Figure 10:**
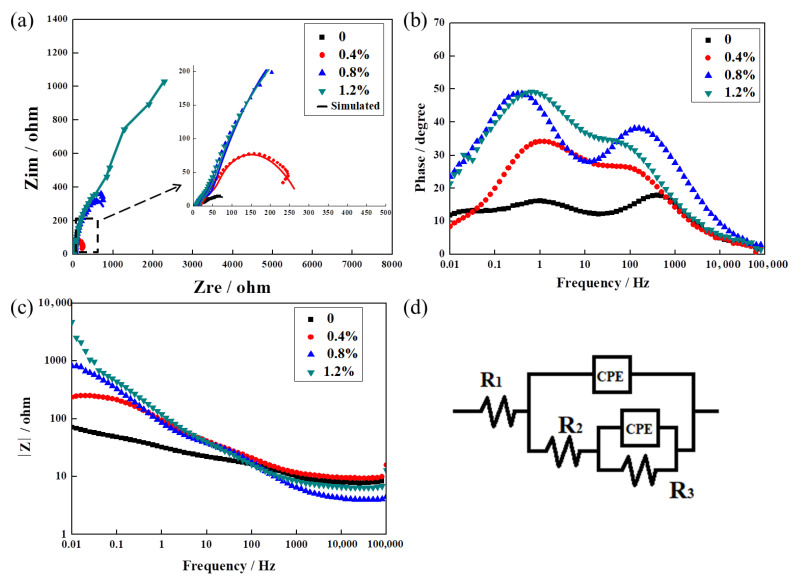
Ag–Cu alloys with different La_2_O_3_ contents in 1% Na_2_S solution—(**a**) EIS Nyquist diagram, (**b**) EIS Bode phase angle plot, (**c**) EIS Bode impedance plot, and (**d**) equivalent circuit diagram used to fit the EIS spectra.

Through the above explanation, the addition of La_2_O_3_ improved the density and hardness of the Ag–Cu samples, which conformed to the first two assumptions of this work. The following content would focus on the corrosion resistance of the Ag–Cu alloy samples containing La_2_O_3_ in sulfide solution. The EIS spectra of the Ag–Cu alloys with different La_2_O_3_ contents in 1% Na_2_S solution are presented in Figure 10, The values obtained from the plots, fitted using ZSimp software (v3.30), are provided in Table 4. According to Figure 10a, the fittings of the equivalent circuit diagram given in Figure 10d to the EIS plots of all the samples were appropriate. The Bode phase angle plots (in Figure 10b) of all the samples presented three valleys, indicating the existence of three time constants in the frequency range. These time constants corresponded to three resistances and two constant phase elements (CPE) connected in parallel. In the equivalent circuit, R_1_ represented the solution resistance, while R_2_ and R_3_ expressed the resistance of the passive film (or the corrosion product layer) and the resistance of the Ag–Cu alloy substrate, respectively. The values listed in Table 4 showed that the resistances of the Ag–Cu alloys containing La_2_O_3_ were enhanced compared to the samples without La_2_O_3_. Furthermore, as the amount of La_2_O_3_ increased, the resistance value of the sample continued to grow. This observation was consistent with the trends shown in Figure 10c. The impedance values of the Ag–Cu alloys with La_2_O_3_ contained improved significantly from ~102 Ω/cm^2^ (the sample without La_2_O_3_) to ~383 Ω/cm^2^ (0.4% La_2_O_3_ content), ~1090 Ω/cm^2^ (0.8% La_2_O_3_ content), and ~4145 Ω/cm^2^ (1.2% La_2_O_3_ content). Higher total impedance values (R_1_, R_2_, and R_3_) indicate greater corrosion resistance.

**Figure 11:**
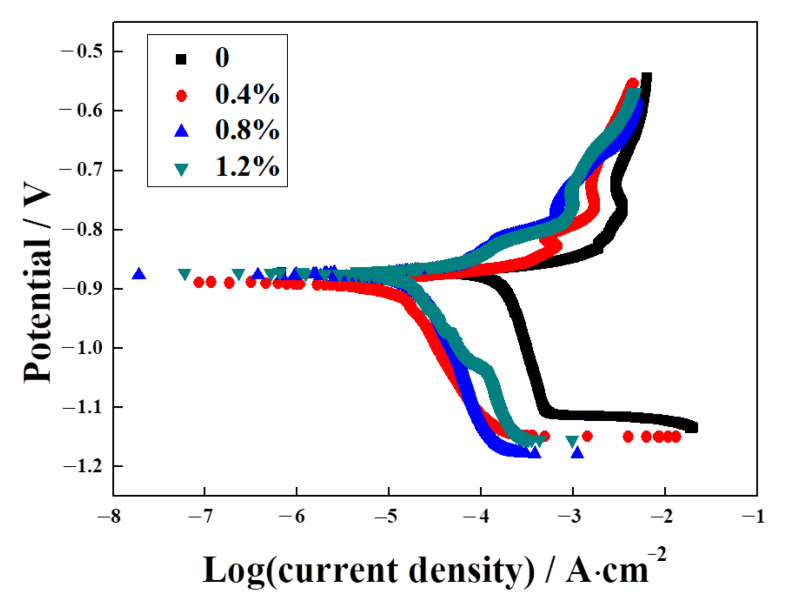
Potentiodynamic polarization curves of Ag–Cu alloys with different La_2_O_3_ contents in 1% Na_2_S solution.

The PD curves of the Ag–Cu alloys with different La_2_O_3_ contents in 1% Na_2_S solution are presented in Figure 11, The values of the corrosion current densities (*I*_corr_) and corrosion potentials (*E*_corr_) extracted from the curves are listed in Table 5. The corrosion potentials of all the samples were similar numerically. Higher corrosion potentials represent a lower corrosion tendency. Thus, in terms of corrosion tendency only, all the samples were very close. While comparing the curve of the sample without La_2_O_3_, it was observed that the curves of all the samples with La_2_O_3_ shifted to the left by varying degrees; this shift resulted in a decrease in their corrosion current densities. It was suggested that the corrosion rate of the Ag–Cu alloys slowed down after La_2_O_3_ was added. A reasonable explanation for this is provided by previous work [4], indicating that there is a higher occurrence of preferential corrosion within the interior grain than along the grain boundary in Ag–Cu alloys. As the La_2_O_3_ content grew, the grain sizes decreased, as shown in Figure 6; this shows that the quantity of grain boundaries increased. Thus, the corrosion rate of the Ag–Cu alloys slowed down after the addition of La_2_O_3_. The values in Table 5 also support the view that as the La_2_O_3_ content increased from 0 to 1.2%, the corrosion current densities decreased gradually; 1.2% La_2_O_3_-containing Ag–Cu alloys achieved the minimum corrosion current density of 9.62 μA∙cm^−2^, which significantly declined from 126.35 μA∙cm^−2^ in the sample without La_2_O_3_. Through EIS and PD tests of the samples in 1% Na_2_S solution, the practical and efficacious effects of La_2_O_3_ addition on the corrosion resistance of Ag–Cu alloys prepared by SLM, in particular their resistance to sulfidation, was demonstrated.

## 4. Conclusions

In this work, to improve the laser absorptivity and to acquire better mechanical properties and higher resistance to sulfidation, Ag–Cu alloys with different La_2_O_3_ contents were prepared using the SLM process, via mechanical mixing of La_2_O_3_ particles with Ag–Cu alloys powders. Then, the phase compositions and microstructures of the Ag–Cu alloys with different La_2_O_3_ contents were analyzed. Additionally, their mechanical properties and corrosion resistance in 1% Na_2_S solution were evaluated. The main conclusions of this work can be summarized as follows:

1. The addition and mixture of La_2_O_3_ nano-particles to Ag–Cu alloy powders plays a positive role in improving laser absorptivity and reducing defects during the SLM process, leading to a significant increase in the density of Ag–Cu alloys, rising from 7.76 g/cm^3^ to 9.16 g/cm^3^.
2. The phase composition of Ag–Cu alloys, as prepared by SLM, was found to be Silver-3C. The presence of La_2_O_3_ had no influence on this composition, but refined the grains of the Ag–Cu alloys by inhibiting the growth of columnar grains during the SLM process. No particular preferred orientation was observed in either the samples prepared with or without La_2_O_3_.
3. There was an upwards trend in the hardness of the Ag–Cu alloys as the La_2_O_3_ content was increased from 0 to 1.2% (mass fraction). The average hardness significantly increased from 0.97 GPa to 2.88 GPa when 1.2% La_2_O_3_ was added, which was also higher than the ~1.11 GPa hardness of the sample with a similar chemical composition prepared by the powder metallurgy method, reported by a previous study [39]. This enhancement in hardness was mostly due to a reduction in pore defects and the refined grains caused by the effects of adding La_2_O_3_.
4. EIS and PD tests of the samples in 1% Na_2_S solution proved that the addition of La_2_O_3_ improved the corrosion resistance of Ag–Cu alloys prepared by SLM practically and efficaciously. This improvement was evident in the form of higher impedance values and lower corrosion current densities.

## Figures and Tables

**Table 1:** Chemical compositions of the Ag–Cu powders used in this work.

Element	Cu	Ge	K	Si	Ca	Ag
Mass fraction (%)	5.626	0.893	0.431	0.242	0.138	Bal.

**Table 2:** SLM process parameters used for preparing the Ag–Cu alloy samples.

Laser Power	Layer Thickness	Scanning Speed	Focus Offset	Oxygen Content
60 W	20 μm	1000 mm/s	0 mm	<0.3%

**Table 3:** The densities of the Ag–Cu samples with different La_2_O_3_ contents.

La_2_O_3_ Content (Mass Fraction)	0%	0.4%	0.8%	1.2%
Density (g/cm^3^)	7.76 ± 0.22	8.14 ± 0.26	8.55 ± 0.33	9.16 ± 0.55

**Table 4:** EIS resistance values of Ag–Cu alloys with different La_2_O_3_ contents in 1% Na_2_S solution.

La_2_O_3_ Content (wt%)	*R*_1_ (Ω/cm^2^)	CPE (10^−4^ F∙cm^2^)	*n*_1_	*R*_2_ (Ω/cm^2^)	CPE (10^−3^ F∙cm^2^)	*n*_2_	*R*_3_ (Ω/cm^2^)
0	7.77 ± 0.73	1.82 ± 0.25	0.82 ± 0.05	7.74 ± 1.02	2.32 ± 0.03	0.38 ± 0.01	86.91 ± 6.65
0.4%	9.90 ± 2.87	0.22 ± 0.03	0.68 ± 0.03	23.44 ± 3.36	1.46 ± 0.05	0.81 ± 0.05	0.35 ± 0.08 × 10^3^
0.8%	9.57 ± 1.43	5.39 ± 0.83	0.71 ± 0.02	39.50 ± 5.56	3.36 ± 0.18	0.73 ± 0.02	1.04 ± 0.21 × 10^3^
1.2%	7.25 ± 1.69	6.54 ± 0.99	0.75 ± 0.06	47.54 ± 9.31	1.64 ± 0.41	0.77 ± 0.01	4.09 ± 0.30 × 10^3^

**Table 5:** Corrosion current density and corrosion potential of Ag–Cu alloys with different La_2_O_3_ contents in 1% Na_2_S solution.

La_2_O_3_ Content (wt%)	0	0.4	0.8	1.2
*E_corr_* (V)	−0.87 ± 0.015	−0.88 ± 0.040	−0.89 ± 0.045	−0.87 ± 0.055
*i_corr_* (μA∙cm^−2^)	126.35 ± 17.33	40.80 ± 2.16	11.23 ± 2.68	9.62 ± 0.89

## Data Availability

Data is contained within the article.
